# Iron Regulatory Protein 1 Inhibits Ferritin Translation Responding to OsHV-1 Infection in Ark Clams, *Scapharca Broughtonii*

**DOI:** 10.3390/cells11060982

**Published:** 2022-03-13

**Authors:** Bowen Huang, Xiang Zhang, Qin Liu, Changming Bai, Chen Li, Chongming Wang, Lusheng Xin

**Affiliations:** 1 Qingdao Key Laboratory of Mariculture Epidemiology and Biosecurity, Key Laboratory of Maricultural Organism Disease Control, Ministry of Agriculture, Yellow Sea Fisheries Research Institute, Chinese Academy of Fishery Sciences, Qingdao 266071, China; huangbwn87@outlook.com (B.H.); zhangxiang01231@outlook.com (X.Z.); baicm@ysfri.ac.cn (C.B.); lichen@ysfri.ac.cn (C.L.); wangcm@ysfri.ac.cn (C.W.); 2 Function Laboratory for Marine Fisheries Science and Food Production Processes, Qingdao National Laboratory for Marine Science and Technology, Qingdao 266071, China; 3 Guangxi Key Laboratory for Agricultural Resources Chemistry and Biotechnology, College of Biology and Pharmacy, Yulin Normal University, Yulin 537000, China; liuqin@ylu.edu.cn

**Keywords:** iron regulatory protein 1, ferritin, iron metabolism, *Scapharca broughtonii*, OsHV-1

## Abstract

Elemental iron is an indispensable prosthetic group of DNA replication relative enzymes. The upregulation of ferritin translation by iron regulatory proteins (IRP1) in host cells is a nutritional immune strategy to sequester available iron to pathogens. The efficient replication of Ostreid herpesvirus 1 (OsHV-1), a lethal dsDNA virus among bivalves, depends on available iron. OsHV-1 infection was found to trigger iron limitation in ark clams; however, it is still an enigma how OsHV-1 successfully conducted rapid replication, escaping host iron limitations. In this study, we identified the IRP1 protein (designated as *Sb*IRP-1) in the ark clam (*Scapharca broughtonii*) and found it could bind to the iron-responsive element (IRE) of ferritin (*Sb*Fn) mRNA based on electrophoretic mobility shift assay (EMSA). Knockdown of *Sb*IRP-1 expression (0.24 ± 1.82-fold of that in NC group, *p* < 0.01) by RNA interference resulted in the accumulation of *Sb*Fn in hemocytes (1.79 ± 0.01-fold, *p* < 0.01) post-24 h of enhanced RNA interference injection. During OsHV-1 infection, *Sb*Fn mRNA was significantly upregulated in hemocytes from 24 h to 60 h, while its protein level was significantly reduced from 24 h to 48 h, with the lowest value at 36 h post-infection (0.11 ± 0.01-fold, *p* < 0.01). Further analysis by RNA immunoprecipitation assays showed that OsHV-1 could enhance the binding of *Sb*IRP-1 with the *Sb*Fn IRE, which was significantly increased (2.17 ± 0.25-fold, *p* < 0.01) at 36 h post-infection. Consistently, *Sb*IRP-1 protein expression was significantly increased in hemocytes from 12 h to 48 h post OsHV-1 infection (*p* < 0.01). In conclusion, the results suggest that OsHV-1 infection could suppress post-transcriptional translation of *Sb*Fn through the regulation of *Sb*IRP-1, which likely contributes to OsHV-1 evasion of *Sb*Fn-mediating host iron limitation.

## 1. Introduction

Iron performs a critical role in cellular metabolism as a component of prosthetic groups in electron transport proteins and various enzymes involved in DNA synthesis, ATP production, etc. [1,2]. Virus replication is highly dependent on the host cell, and its efficient replication requires an available iron-rich microenvironment [2,3]. A virus with a life cycle of DNA stages requires dNTPs provided by ribonucleotide reductase (RR), which contains an iron tyrosyl radical center essential for activity [4,5]. In a previous study, the membrane permeable elater (2,2’-bipyridine) was found to inhibit the activation of vaccinia virus RR and strongly inhibited viral replication [6]. To ensure that infected cells are enriched with iron and favor viral rapid replication, some viruses selectively infect iron-rich cells by targeting transferrin receptor 1 (TfR1) [7] or disrupt host iron homeostasis by interfering with the regulation of host iron-metabolism-related proteins, such as hepcidin, MHC class I-like protein HFE (Homeostatic Iron Regulator), TfR1, and ferritin [8,9,10,11,12]. Similarly, pathogens such as bacteria and fungi have also parallelly evolved various strategies against iron limitation in hosts, such as secreting siderophores to compete for available iron in the host, or directly acquiring host iron via heme/hemoprotein receptors [2,13].

Ferritin complex has a hollow nanocage capable of storing up to 4500 iron atoms, is involved in iron metabolism, and widely exists in organisms [14,15]. The majority of cellular iron is sequestered and stored in ferritins in a non-toxic but bioavailable form [16]. Cellular ferritin levels are mainly regulated post-transcriptionally by iron regulatory proteins (IRP1 and IRP2), which could anchor to the iron response element (IRE) in the 5’ untranslated region (UTR) of the ferritin mRNA [17]. IRP1 is a bifunctional protein which predominantly responds to intracellular iron levels by assembling/disassembling the [4Fe-4S] cluster to switch between aconitase (closed conformation) and trans-regulated forms (open conformation) [18]. IRP2 is homologous to IRP1, but functions without the [4Fe-4S] cluster. The content of IRP2 is regulated at the protein level and is rapidly degraded by the proteasome in iron-replete cells [19]. Low levels of cellular iron promote the binding of IRPs to the IRE in the 5’ UTR of ferritin mRNA, leading to the downregulation of ferritin and the accumulation of free iron. When cellular iron is excessive, it will promote the assembly of IRP1 with the [4Fe-4S] cluster, switching from the IRE-binding form to the aconitase, and trigger proteasomal degradation of IRP2 to facilitate the translation of ferritin [19]. Additionally, the expression of ferritin is also regulated at the transcriptional level through the activation of transcription factors including nuclear-factor-E2related factor 2 and nuclear factor kappa B [20]. Both oxidative stress and inflammation were reported to promote the transcription of ferritin genes [21].

The IRE is conservatively found in the 5’UTR of the ferritin mRNA in invertebrates [22]. IRP1 homologues with IRE-binding activity have been identified in various invertebrates such as *Drosophila melanogaster* [23], *Aedes aegypti* [24], crayfish *Pacifastacus leniusculus* [25], and the earthworms *Eisenia andrei* [26]. Recombinant *Manduca sexta* IRP1 was found to inhibit the in vitro translation of ferritin mRNA through interactions with the IRE [27]. Meanwhile, iron-dependent translational control through IRE/IRP interactions is verified in *M. sexta*. Low concentrations of iron administration reduced the binding activity of *M. sexta* fat body IRP1 with the IRE and resulted in increased levels of ferritin [28]. In addition to the direct response to cellular iron concentrations, the IRE/IRP interaction in invertebrates was also affected by immune challenge. Mosquito cells exhibited an increase in IRP1/IRE binding activity when they were exposed to lipopolysaccharide [24].

OsHV-1, as a linear double-stranded DNA virus, belongs to the *Malacoherpesviridae* family and becomes a main epidemic virus pathogen among bivalves [29,30]. The transcriptome of OsHV-1 revealed high levels of DNA polymerase and RR transcripts consistent with high viral loads in the host [31]. High expression of these DNA replication-related enzymes is likely to be critical for the rapid progression of infection and it is notable that all these enzymes require iron to function [32]. Our previous studies demonstrated that in vivo, the iron microenvironment was disturbed in OsHV-1-infected ark clams, and the expression pattern of *Sb*Fn was dramatically affected post-virus infection [33]. To further elucidate how OsHV-1 influences host cellular iron homeostasis, the potential regulatory role of IRPs on cellular iron homeostasis was investigated in OsHV-1-infected ark clams. Firstly, an IRP1 member (*Sb*IRP-1) was identified from ark clams (*S.broughtonii*) whose mRNA expression pattern was detected post-OsHV-1. Meanwhile, the regulatory function of *Sb*IRP-1 was further surveyed. The results indicated the regulatory role of the *Sb*IRP-1-*Sb*Fn pathway on iron homeostasis.

## 2. Materials and Methods

### 2.1. Animals and Challenge Experiment

The Healthy adult ark clams (two years old) were collected from a local farm in Rizhao (Shandong Province, China). Before processing, the ark clams were acclimatized for one week in natural seawater supplied with oxygen at 18 °C (20 ark clams in each 40 L tank). For experimental infections, OsHV-1 suspension was firstly prepared as previously described, with modifications [34]. Briefly, mantle tissues of OsHV-1-infected ark clams were collected and homogenized. After short centrifugation at 1000 rpm, 4 °C, the supernatant was filtered sequentially using filters with pore sizes of 5 μm, 2 μm, 0.45 μm, and 0.22 μm and stored at 4 °C before use. One hundred ark clams were randomly divided into five groups. Four groups of ark clams were injected with 100 μL OsHV-1 suspension (~10^6^ copies of viral DNA/μL), while the remaining group was injected with the same volume of negative tissue homogenate as the control. For RNA extraction, triple tissue samples of hemocytes were collected respectively from three ark clams at 0 h, 12 h, 24 h, 36 h, 48 h and 60 h post injection. Different tissues, including mantle, gill, adductor muscle, foot, hemocyte, and hepatopancreas were obtained from untreated ark clams by using sterilized scissors and tweezers. All samples were added with 1 mL TRIzol reagent (Invitrogen, Waltham, MA, USA) and stored at −80 °C before RNA extraction.

### 2.2. Total RNA Extraction and cDNA Synthesis

Total RNA was extracted from 50 mg of each tissue sample using TRIzol reagent (Invitrogen) according to the manufacturer’s protocol. The first strand of cDNA was synthesized using the template of DNase I-treated total RNA according to the manufacturer’s protocol (TaKaRa, Shiga, Japan). The synthesis reaction was processed at 37 °C for 20 min and stopped by heating at 85 °C for 5 s. The cDNA mixture was diluted to 1:40 and stored at −80 °C.

### 2.3. Clone and Bioinformatic Analyses of SbIRP-1

The specific primers *Sb*IRP-F and *Sb*IRP-R (Table 1) were designed by Primer Premier 5 software to clone the full sequence of *Sb*IRP-1 based on the sequence information of ark clam genomic data [35]. Then, the above PCR product was purified and cloned into the pMD 19-T simple vector (TaKaRa) and sequenced.

For characterization of the *Sb*IRP-1 sequence, the amino acid sequences of *Sb*IRP-1 were deduced by the ExPASy Protein Analysis System v3.0. http://www.expasy.org/ (accessed on 15 December 2021) and comparatively analyzed by the BLAST algorithm http://www.ncbi.nlm.nih.gov/blast (accessed on 16 December 2021). The protein domains were revealed by SMART v9.0. http://smart.embl-heidelberg.de/ (accessed on 16 December 2021). Multiple sequence alignment of *Sb*IRP-1 with IRPs of other species was performed by the ClustalX multiple alignment program (Conway Institute UCD Dublin, Dublin, Ireland) and the result was generated in ESPript v3.0. https://espript.ibcp.fr/ESPript/cgi-bin/ESPript.cgi (accessed on 20 December 2021). A phylogenetic tree of IRPs was constructed by the neighbor-joining algorithm using the Mega program v6.0. (Tokyo Metropolitan University, Tokyo, Japan), and 1000 bootstrap replicates were performed. The three-dimensional structures of IRPs were constructed using SWISS-MODEL https://swissmodel.expasy.org (accessed on 21 December 2021) and showed by PyMol program v2.3.0. (Schrödinger, New York, NY, USA).

### 2.4. RT-qPCR Analysis of SbIRP-1 and SbFn mRNA

The expression levels of *Sb*IRP-1 and *Sb*Fn were measured by SYBR RT-qPCR reagent (TOYOBO, Osaka, Japan) based on Bio-Rad CFX Connect real-time PCR system. Specific primers, q*Sb*IRP-F, and q*Sb*IRP-R (Table 1) were used for amplifying a 172 bp fragment of *Sb*IRP-1, q*Sb*Fn-F, and q*Sb*Fn-R (Table 1) for a 153 bp fragment of *Sb*Fn. 60S ribosomal protein subunit fragment, amplified with primers q*Sb*RL15-F and q*Sb*RL15-R (Table 1), was chosen as an internal reference. The relative expression level of mRNA was analyzed by the 2^−^^ΔΔCt^ method [36].

### 2.5. SbIRP-1 Recombinant Protein Expression and Polyclonal Antibody Preparation

The vector construction and expression of recombinant *Sb*IRP-1 were performed as previously described [37]. The coding region of *Sb*IRP-1 was amplified with the primers *Sb*IRP-*Nco* I and *Sb*IRP-*Xho* I (Table 1). The amplified fragments were purified and digested with *Nco* I and *Xho* I before being inserted into the same double restriction enzyme linearized expression vector pET-28a. The constructed vector pET-28a-*Sb*IRP-1 was transferred into BL21 (DE3) Chemically Competent Cell (TransGen, Beijing, China). Transformed cells were induced with 0.2 mM IPTG and cultured overnight at 18 °C. Histidine-labeled r*Sb*IRP-1 was purified using ProteinPure Ni-NTA resin (TransGen) and concentrated by ultrafiltration after dialysis. For polyclonal antibody preparation, 2 mg of r*Sb*IRP-1 protein was used as an antigen mixed thoroughly with an equal volume of Freund’s complete adjuvant and injected into New Zealand white rabbit at the first immunization, which was followed by a second and third immunization with 2 mg of r*Sb*IRP-1 protein and the same volume of Freund’s incomplete adjuvant at 10-day intervals. The serum was harvested and stored at −80 °C before used. The specificity of the *Sb*IRP-1 polyclonal antibody was tested by western blot of the r*Sb*IRP-1 and hemocyte lysate.

### 2.6. The Knockdown of SbIRP-1

Small interfering RNA (si*Sb*IRP) specific to *Sb*IRP-1 and negative control (siNC) were designed and synthesized by Sangon Biotech (Shanghai, China) (Table 1). For RNA interference, si*Sb*IRP (100 μL, 0.1 μg per μL) and siNC (100 μL, 0.1 μg per μL) were injected into the ark clams of experimental and control groups, respectively. To enhance the effect of RNA interference, a second injection was performed 12 h after the first injection using the same dosage. Hemocytes from the experimental and control groups were sampled 24 h after the second injection for further analysis of RNA and protein levels of *Sb*IRP-1.

### 2.7. Electrophoretic Mobility Shift Assay

To test the binding activity of *Sb*IRP-1 to *Sb*Fn-IRE, EMSA was performed using a chemiluminescent EMSA kit (Beyotime, Shanghai, China) according to the manufacturer’s instructions. IREs were predicted using SIREs web server v2.0. http://ccbg.imppc.org/sires/ (accessed on 10 October 2021) and RNAfold Web Server http://www.unafold.org/ (accessed on 24 November 2021). Biotin-labeled *Sb*Fn-IRE probes and mutation *Sb*Fn-IRE probes, and unlabeled *Sb*Fn-IRE probes were synthesized by Sangon Biotech (Shanghai, China). The sequences of the EMSA probes were listed in Table 1. For EMSA, the probes were heated at 95 °C for 3 min, then slowly cooled to room temperature, annealed to form a stem-loop structure, and then incubated with 1 μg of r*Sb*IRP-1 for 30 min at 25 °C. Cold competitor probes (unlabeled SbFn-IRE probes) at 20×, 10× and 5× were used to confirm the binding specificity. Samples were separated using 6% native polyacrylamide gel, then transferred to nylon membrane and crosslinking by UV. Transferred probes were detected with HRP-conjugated streptavidin.

### 2.8. RNA Immunoprecipitation

RNAChIP was performed using the RNA ChIP-IT kit (Active Motif, La Hulpe, Belgium) according to the manufacturer’s instructions. Briefly, approximately 1 × 10^7^ hemocytes were cross-linked with 1% glutaraldehyde for 10 min at room temperature and terminated with 125 mM glycine. Cells were lysed on ice for 30 min using ice-cold NP40 lysis buffer containing protease inhibitor cocktail (Roche, Switzerland) and RNase Inhibitors (TaKaRa). Cell lysates were digested using DNase I before the immunoprecipitation reaction; a portion of treated cell lysates was saved as input RNA template at −80 °C before use. Immunoprecipitation was performed using *Sb*IRP-1 polyclonal antibody or normal rabbit IgG (ABclonal, Wuhan, China) as control with equal amounts of DNase I-treated cell lysates overnight at 4 °C, respectively. RNA binding with *Sb*IRP-1 was eluted from the magnetic beads, reversely cross-linked, and purified using TRIzol reagent (Invitrogen) according to the manufacturer’s protocol. The first-strand cDNA of purified RNA was synthesized using PrimeScript™ RT reagent Kit with gDNA Eraser (TaKaRa) and quantified by qPCR. Data were normalized to the corresponding RNA input. The sequence information of the primers was listed in Table 1.

### 2.9. Western Blot

Total protein of the hemocytes was extracted using the cell lysis buffer (Beyotime) containing a protease inhibitor cocktail (Roche). The protein concentration was quantified using a BCA protein assay kit (Transgen). The extracted protein samples were separated using 12% SDS-PAGE and transferred onto nitrocellulose (NC) membranes. After blocking with 5% skimmed milk powder, membranes were incubated overnight at 4 °C with *Sb*IRP-1 polyclonal antibody (1:500), *Sb*Fn (1:500) polyclonal antibody [33] or *Sb*Tubulin polyclonal antibody (1:1000). Membranes were then incubated with HRP-conjugated goat anti-mouse Ig and goat anti-rabbit Ig secondary antibodies (1:3000, Abclonal) for 3 h at room temperature with shaking. Between each step, the NC membranes were washed thrice for 5 min with TBST. Protein bands were visualized in an automated chemiluminescent gel imaging system using Western lighting ECL substrate (Thermo Fisher Scientific, Waltham, MA, USA).

### 2.10. Statistical Analysis

All experiments were repeated thrice independently, and the data are presented as mean ± SD. Statistical analysis of the data was carried out using the Statistical Package for Social Sciences (SPSS) 21.0. Differences between groups were assessed using one-way ANOVA. Differences between means are considered significant at *p* < 0.05 and extremely significant at *p* < 0.01.

## 3. Results

### 3.1. Molecular Characterization of SbIRP-1 Statistical Analysis

**Figure 1:**
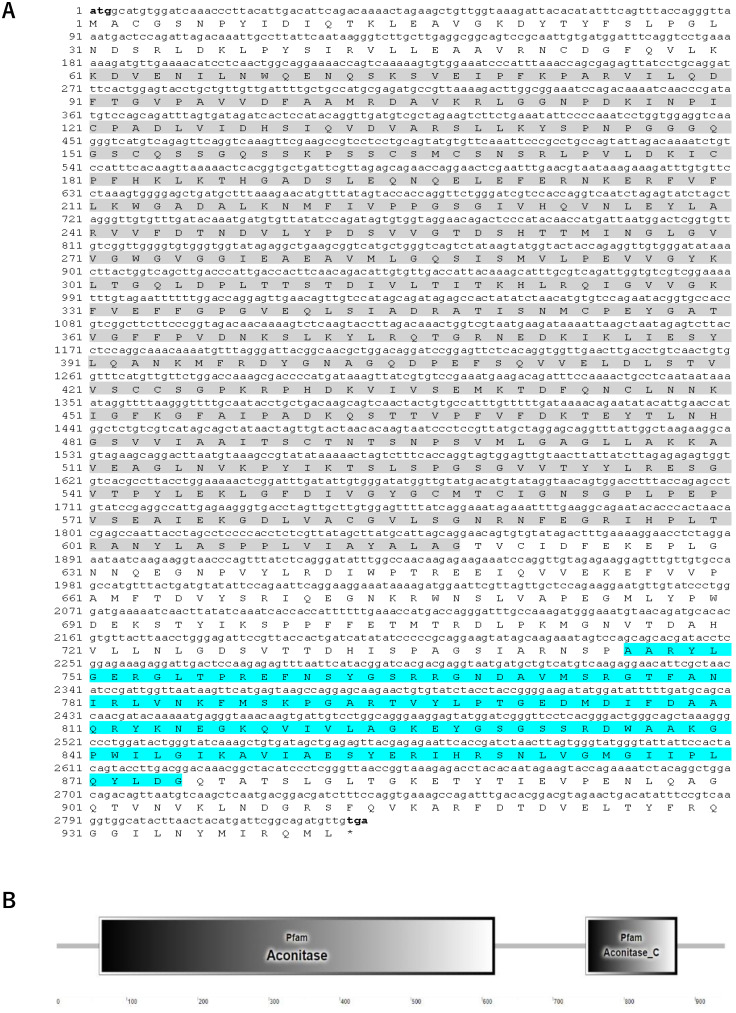
Sequence characterization of *Sb*IRP-1. (**A**) Nucleotide and deduced amino acid sequences of *Sb*IRP-1. Nucleotides and amino acids are numbered along the right margin. The initiation codon (atg) and a termination codon (tga) are presented in bold. The aconitase domain is marked in gray and the aconitase C-terminal domain is marked in cyan. (**B**) Protein domains of *Sb*IRP-1 predicted by SMART.

**Figure 2:**
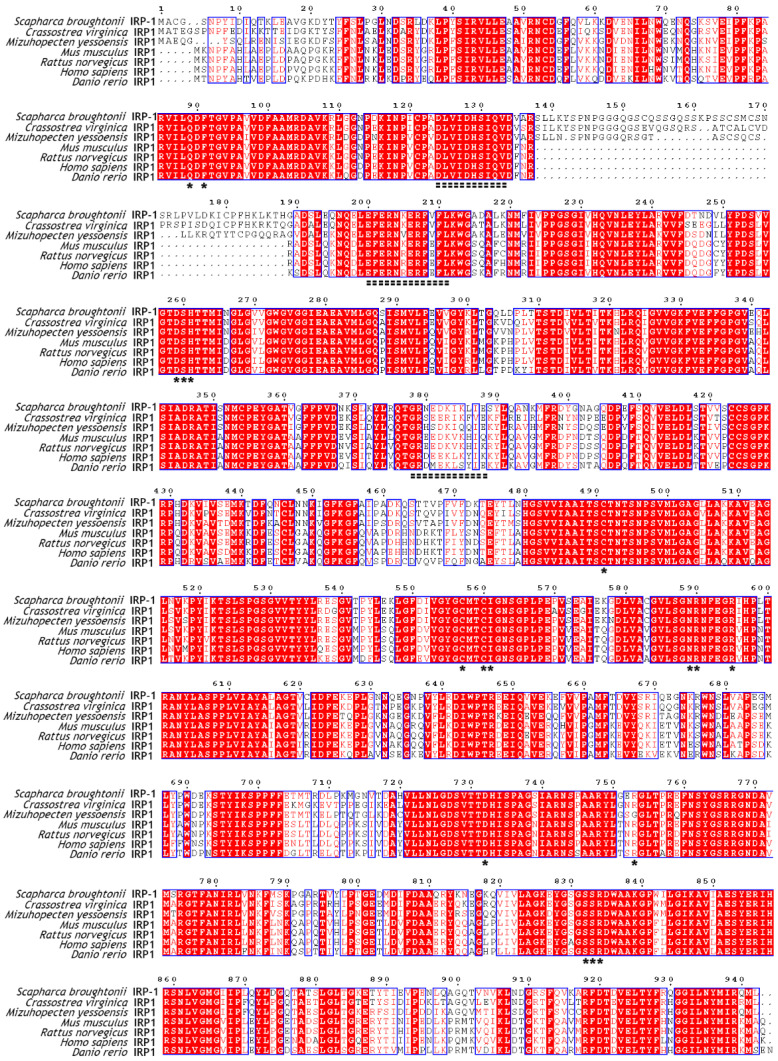
Multiple sequence alignment of *Sb*IRP-1 with other IRP1s from various species. The amino acid number above the sequence refers to *Sb*IRP-1. Asterisks represent active sites in the aconitase domains. The dashed amino acid fragments indicate putative RNA binding regions. Red shading regions indicate similar (consensus >70%) amino acids. The species (access number) include *Crassostrea virginica* (XP_022311890.1), *Mizuhopecten yessoensis* (XP_021340677.1), *Mus musculus* (NP_031412.2), *Rattus norvegicus* (NP_059017.2), *Homo sapiens* (NP_001265281.1), *Danio rerio* (NP_001030155.1).

The full-length cDNA of *Sb*IRP-1 with an ORF of 2829 bp encodes a putative protein of 942 amino acids (with a predicted molecular mass of 103.3 kDa and a theoretical pI of 6.16) (Figure 1A). SMART prediction domain analysis showed that *Sb*IRP-1 contained conservative aconitase (Lys^61^-Gly^618^) and aconitase C-terminal (Ala^746^-Gly^875^) domains (Figure 1B). Multiple sequence comparisons showed that *Sb*IRP-1 possessed 83% similarity to IRP1 form *Crassostrea virginica* (XP_022311890.1) and 69% similarity to IRP1 form *Homo sapiens* (NP_001265281.1), but the aconitase domain (Ser^138^-Gly^189^) of *Sb*IRP-1 exhibited low homology with those of other molluscan IRP1s and was absent in vertebrate IRP1s (Figure 2). Conserved active sites were found in *Sb*IRP-1, including three [4Fe-4S] cluster-ligating cysteine residues (Cys^491^, Cys^557^, and Cys^560^), which performed essential roles in iron regulation of RNA binding and aconitase activity, and four arginine residues (Arg^590^, Arg^595^, Arg^753^, and Arg^834^) in the aconitase active-site cleft were essential for RNA recognition. Additionally, three putative RNA binding regions (^124^DLVIDHSIQV^133^) [38], (^200^EFERNKERFVFL^211^) and (^378^GRNEDKIKLIE^388^) [39] were also conserved in the *Sb*IRP-1.

**Figure 3:**
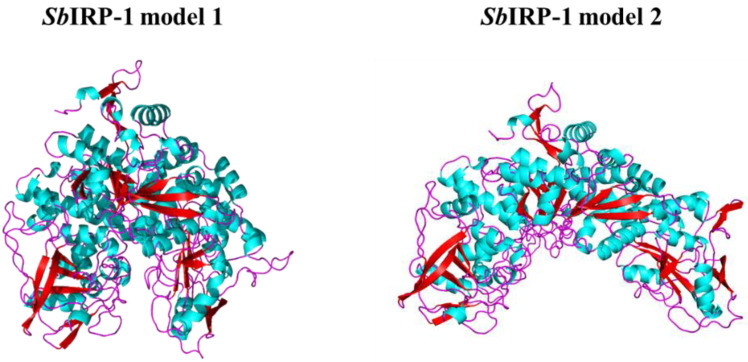
The three-dimensional structures of *Sb*IRP-1. The structures were predicted by using SWISS-MODEL and displayed by PyMol. The *α*-helix structure is marked in cyan, the *β*-sheet structure in red, and the L-ring in magenta. *Sb*IRP-1 model 1: assembling with the [4Fe-4S] cluster; model 2: disassembling with the [4Fe-4S] cluster.

The three-dimensional structure of *Sb*IRP-1 was calculated using the SWISS-MODEL program. The results revealed two alternative conformations of *Sb*IRP-1; model-template alignments showed that *Sb*IRP-1 (amino acids 5–941) shared 72.57% sequence identity with the human cytosolic aconitase (IRP1) model 1 (PDB ID: 2b3y.1.A) assembling with the [4Fe-4S] cluster, and 73.25% sequence identity with the rabbit cytosolic aconitase (IRP1) model 2 (PDB ID: 3sn2.1.A) disassembling with the [4Fe-4S] cluster. The QMEAN Z-scores for the structures of *Sb*IRP-1 obtained based on the homology modeling of the above two models were −1.22 and −1.90, respectively, indicating that the proposed homology model is reliable and acceptable [40] (Figure 3).

**Figure 4:**
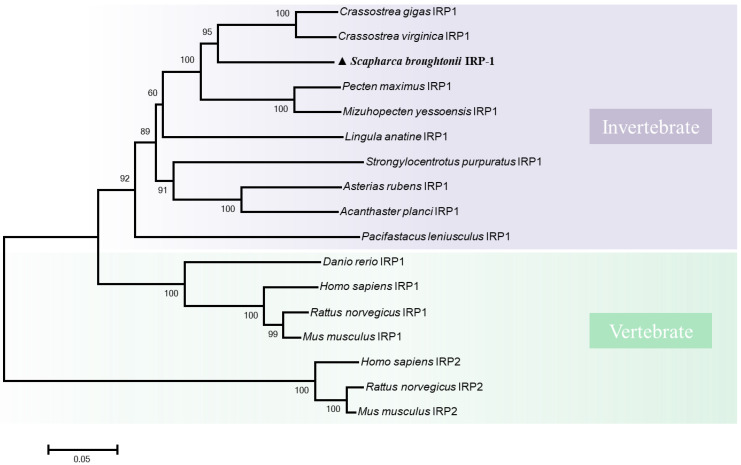
Phylogenetic tree of *Sb*IRP-1 and other known IRP family members. The tree was obtained using MEGA 6.0 with the neighbor-joining method. The numbers at the branches indicate the bootstrap value for 1,000 replicates. The accession numbers of these proteins obtained from GenBank are as follows: *Crassostrea gigas* IRP1 (XP_034321625.1), *C.virginica* IRP1 (XP_022311890.1), *Pecten maximus* IRP1 (XP_033725425.1), *M.yessoensis* IRP1 (XP_021340677.1), *Lingula anatine* IRP1 (XP_013409788.1), *Strongylocentrotus purpuratus* IRP1 (XP_030855986.1), *Asterias rubens* IRP1 (XP_033624337.1), *Acanthaster planci* IRP1 (XP_022100487.1), *Pacifastacus leniusculus* IRP1 (CAB41634.1), *D.rerio* IRP1 (NP_001030155.1), *H.sapiens* IRP1 (NP_001265281.1), *R.norvegicus* IRP1 (NP_059017.2), *M.musculus* IRP1 (NP_031412.2), *H.sapiens* IRP2 (NP_004127.2), *R.norvegicus* IRP2 (NP_074054.2), *M.musculus* IRP2 (NP_073146.2).

The phylogenetic tree of the IRPs was shown in Figure 4. IRP1 proteins from vertebrates (including fish and mammals) and invertebrates are clustered together forming an independent branch. The other branch consisted of three IRP2 proteins from vertebrates. Thereinto, *Sb*IRP-1 is more closely clustered with the other invertebrate IRP1 proteins.

### 3.2. Tissue Distribution and Expression Profile Post OsHV-1 Infection of SbIRP-1

**Figure 5:**
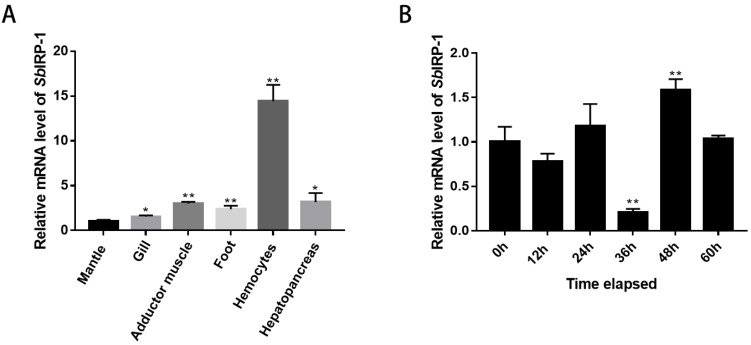
Tissue distribution and expression profile post-OsHV-1 infection of *Sb*IRP-1. (**A**) Tissues distribution of *Sb*IRP-1 in healthy ark clams detected by qRT-PCR. The transcript levels in the mantle, gill, adductor muscle, foot, hemocytes and hepatopancreas were normalized to that in the mantle. (**B**) Expression profiles of *Sb*IRP-1 mRNA after OsHV-1 infection in the hemocytes. Vertical bars represent the means ± SD (*n* = 3). Asterisks indicate significant differences: ** *p* < 0.01, * *p* < 0.05.

The spatial expression of the *Sb*IRP-1 gene in the mantle, gills, adductor muscle, foot, hemocytes, and hepatopancreas were detected by qPCR, which revealed significant differences in the mRNA expression levels of the *Sb*IRP-1 gene among various tissues. The mRNA expression level of the *Sb*IRP-1 gene was the highest in hemocytes (14.43 ± 1.82-fold, *p* < 0.01) among all detected tissues, followed by the hepatopancreas (3.16 ± 1.01-fold, *p* < 0.05) and the adductor muscle (3.01 ± 0.18-fold, *p* < 0.01) (Figure 5A).

*Sb*IRP-1 mRNA expression level was strongly affected by OsHV-1 infection. The mRNA expression level of *Sb*IRP-1 genes in hemocytes significantly increased (1.58 ± 0.12-fold, *p* < 0.01) at 48 h post-OsHV-1 infection (Figure 5B).

### 3.3. Recombinant Expression, Purification and Antiserum Preparation of SbIRP-1

**Figure 6:**
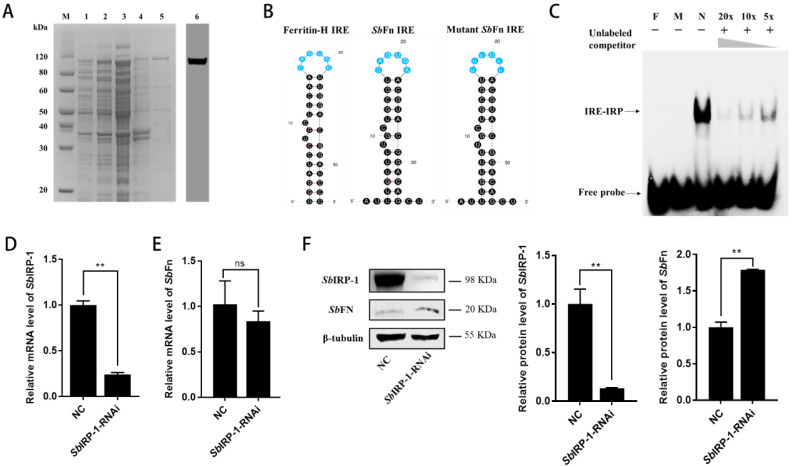
The regulatory role of *Sb*IRP-1 on the translation of *Sb*Fn. (**A**) The recombination protein of *Sb*IRP-1 and specificity of its polyclonal antibody. M: protein marker; Lane 1: BL21 harboring pET-28a-*Sb*IRP-1 (without induction); Lane 2: IPTG-induced BL21 harboring pET-28a-*Sb*IRP-1; Lane 3: supernatant of IPTG-induced bacteria lysate; Lane 4: precipitation of IPTG-induced bacteria lysate; Lane 5, purified r*Sb*IRP-1; Lane 6, Western blot analysis of hemocyte lysates using *Sb*IRP-1 polyclonal antibody. (**B**) Putative structures of consensus iron response elements (IREs) located on the 5’-UTR of human-ferritin-heavy chain mRNA and *Sb*Fn mRNA, and mutant *Sb*Fn IRE used in this study. (**C**) Competitive EMSA, biotin-labeled *Sb*Fn IRE probe was incubated with increasing concentration (20×, 10× and 5×) of cold competitor probe (unlabeled *Sb*Fn IRE probe, shown as the triangle). F indicates free *Sb*Fn IRE probe, M indicates mutant *Sb*Fn IRE probe, and N indicates no cold competitor probe added. (**D**) The RNA interference efficiency of *Sb*IRP-1 in hemocytes was analyzed by qRT-PCR. (**E**) The change in relative mRNA expression level of *Sb*Fn after *Sb*IRP-1 knockdown. (**F**) The change in relative protein expression level of *Sb*IRP-1 and *Sb*Fn after *Sb*IRP-1 knockdown by Western blot and relative protein expression levels were analyzed by calculating the density of target protein/β-tubulin. Asterisks indicate significant differences: ** *p* < 0.01; ns, no statistically significant differences.

After IPTG induction, one major protein with an apparent molecular weight of around 100 kDa was detected (Figure 6A, Lane 1, 2), which was consistent with the predicted molecular mass of *Sb*IRP-1. After purification, a single band of about 110 kDa representing r*Sb*IRP-1 was observed in SDS-PAGE (Figure 6A, Lane 5). Western blotting assay of the hemocyte sample with *Sb*IRP-1 polyclonal antibodies revealed that there was a distinct band of about 110 kDa (Figure 6A, Lane 6), which corresponded to the predicted molecular weight of *Sb*IRP-1.

### 3.4. The Role of SbIRP-1 on the Post-Transcriptional Regulation of SbFn

IRPs mediate cellular post-transcriptional iron regulation by interacting with IREs located in the 5′-UTR mRNA of target genes. The 5′-UTR of *Sb*Fn mRNA was found equipped with a stem-loop structure resembling the IRE of the human-ferritin-heavy chain mRNA, which was located 95 bp downstream of the transcription start site. The IRE of *Sb*Fn mRNA showed a conserved six-membered loop sequence 5′ CAGUGN 3′ and a bulging C between the upper and lower stems (Figure 6B). To elucidate whether *Sb*IRP-1 binds to the putative IRE of *Sb*Fn, electrophoretic mobility shift assay (EMSA) was performed using purified recombinant *Sb*IRP-1. As shown in Figure 6C, the lagging band (containing IRE/IRP complex) only appeared in the lane with the IRE of *Sb*Fn, but not with the mutant IRE (AUUUGUUUUGUUGCGUuuuUuuACGUACGGACAGCU), with lowercase letters in the sequence representing mutation sites. To further confirm the specific binding between r*Sb*IRP-1 and the *Sb*Fn IRE, we performed a competitive EMSA analysis using unlabeled *Sb*Fn IRE as competitive probes. The results showed that unlabeled *Sb*Fn IRE can compete with labeled *Sb*Fn IRE, showing decreased label signals with incremental unlabeled *Sb*Fn IRE (Figure 6C).

To further confirm the post-transcriptional regulatory role of the IRE/IRP system on *Sb*Fn protein levels in hemocytes, the RNAi (RNA interference) assay was carried out. After *Sb*IRP-1 was knocked down by RNAi, the mRNA level of *Sb*IRP-1 in the hemocytes decreased significantly (0.24 ± 1.82-fold of that in NC group, *p* < 0.01) post-24 h of enhanced RNA interference injection (Figure 6D). The protein level of *Sb*IRP-1 was also significantly downregulated, which was 0.13-fold of that in NC group (*p* < 0.01) (Figure 6F). Meanwhile, the mRNA level of *Sb*Fn in *Sb*IRP-1-RNAi ark clams showed no significant difference compared with the NC group (Figure 6E), while the protein level of *Sb*Fn increased significantly (1.79 ± 0.01-fold of that in NC group, *p* < 0.01) (Figure 6F).

### 3.5. The Blocking Effect of SbIRP-1 on the Translation of SbFn Post OsHV-1 Infection

**Figure 7:**
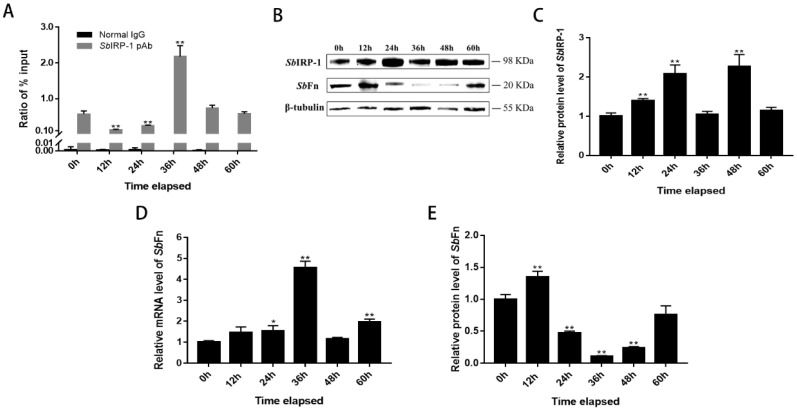
*Sb*IRP-1 pulled the translation of *Sb*Fn down post-OsHV-1 infection. (**A**) Immunoprecipitation of mRNA binding to r*Sb*IRP from hemocytes post-OsHV-1 infection. *Sb*IRP-1 polyclonal antibody and normal rabbit IgG (abclonal) were used to immuno-precipitate RNA binding to r*Sb*IRP-1 and unspecific binding RNA, respectively. The level of *Sb*Fn mRNA binding to r*Sb*IRP-1 was detected by qRT-PCR. **(B**) The protein level of (**C**) *Sb*IRP-1 and (**E**) *Sb*Fn in hemocytes post-OsHV-1 infection was analyzed using Western blot and the relative protein expression level was analyzed by calculating the density of target protein/β-tubulin. (**D**) Expression profiles of *Sb*Fn mRNA post OsHV-1 infection in the hemocytes. Asterisks indicate significant differences: ** *p* < 0.01, * *p* < 0.05.

To evaluate the regulatory role of *Sb*IRP-1 on cellular iron homeostasis during OsHV-1 infection, we initially examined the influence of OsHV-1 infection on the binding ability of *Sb*IRP-1 with the IRE of *Sb*Fn by ChIP-qPCR (Figure 7A). The binding ability of *Sb*IRP-1 with *Sb*Fn IRE was firstly reduced at 12 h and 24 h, then was significantly upregulated at 36 h post-OsHV-1 infection (380.74%, *p* < 0.01), then returned to normal levels. Meanwhile, the protein levels of *Sb*IRP-1 were also examined by western blot post-OsHV-1 infection (Figure 7C). The protein level of *Sb*IRP-1 in hemocytes showed a constant increase from 12 h post-infection (1.39 ± 0.05-fold, *p* < 0.01) to 48 h (2.26 ± 0.31-fold, *p* < 0.01).

OsHV-1 infection significantly promoted the mRNA expression levels *Sb*Fn in hemocytes, which showed significant increase at 24 h (1.54 ± 0.20-fold, *p* < 0.05) and reached a maximum at 36 h post infection (4.55 ± 0.30-fold, *p* < 0.01) (Figure 7D). While the Western blot showed that OsHV-1 infection downregulated *Sb*Fn protein levels (Figure 7E), which were significantly reduced in hemocytes at 36 h (0.11 ± 0.01-fold, *p* < 0.01) post-infection.

## 4. Discussion

**Figure 8:**
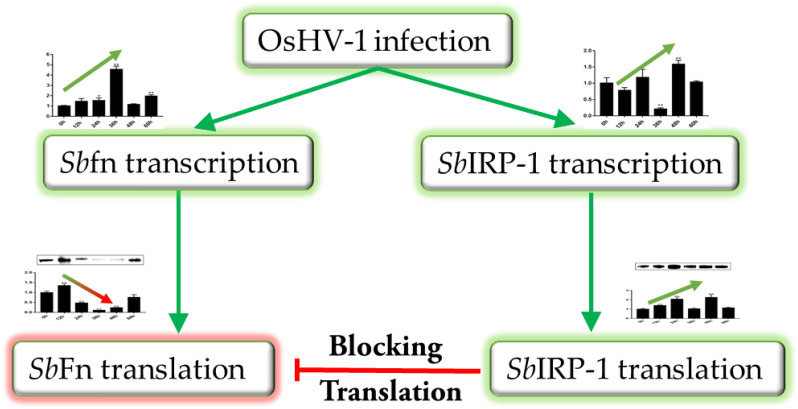
Illustration of the regulatory role of *Sb*IRP-1 on *Sb*Fn post-OsHV-1 infection.

Ferritin is conserved in most organisms as a major intracellular iron storage protein and mainly conducts iron limitation against pathogens [41]. In this study, OsHV-1 infection was shown to promote binding activity of *Sb*IRP-1 to the IRE of *Sb*Fn, blocking the translation of *Sb*Fn, which was intended to reduce the efficient iron sequestration effect of *Sb*Fn, finally facilitating rapid viral replication (Figure 8).

Iron homeostasis in mammalian cells is orchestrated post-transcriptionally by the IRE/IRP system [42]. IRE/IRP1 interaction directly responds to changes in cellular iron content; IRE-binding activity of IRPs is high in iron-deficient conditions and low in iron-sufficient conditions [43]. Here, an IRP1 protein in ark clams (*Sb*IRP-1) was found with an open and a closed conformation, while IRP2 was missing in the genome of ark clams [35]. Moreover, *Sb*IRP-1 possesses three putative RNA binding domains and aconitase active sites, implying the versatile role of *Sb*IRP-1 as RNA binding molecule and aconitase. The IRE was conservatively identified in the 5’-UTR of *Sb*Fn mRNA, resembling ferritin genes across invertebrates, including mollusks, such as housefly (*Musca domestica*), prawn (*Macrobrachium nipponense*), scallop (*Chlamys nobilis*), clam (*Ruditapes decussatus*), abalone (*Haliotis rufescens*), and mussel (*Perna viridis*) [44,45,46,47,48,49]. The location of the *Sb*Fn IRE satisfied the requirement for effective translation inhibition, with a total of 20–30 nucleotides located 90 bp downstream of the transcription start site [50,51]. The IRE/IRP system served conservatively to regulate iron homeostasis in ark clams and a high affinity between r*Sb*IRP-1 and the IRE of *Sb*Fn was revealed by EMSA. When *Sb*IRP-1 was knocked down in vivo by RNAi, the translation of *Sb*Fn was increased and the accumulation level of *Sb*Fn could be detected in hemocytes by western blot, even without significant changes in *Sb*Fn mRNA levels.

OsHV-1, a lethal dsDNA virus, is known as an epidemic viral pathogen in bivalves. Sufficient available iron is necessary for the rapid replication of DNA viruses [5]. Modifications in iron homeostasis have been associated with the pathogenesis of several viruses, including herpes simplex virus, rhabdovirus, hepatitis B virus, and cowpox virus [2,52]. Thus, the association between cellular iron uptake and storage and overall changes in labile iron pool (LIP) may be critical for the development of productive viral infections and pathological situations. Here, it was found that OsHV-1 infection promotes the RNA-binding of *Sb*IRP-1 to *Sb*Fn IRE in ark clam hemocytes. During the later phase of OsHV-1 infection, the binding activity of *Sb*IRP-1 to *Sb*Fn IRE is significantly upregulated, concomitant with a decrease in *Sb*Fn mRNA translation. A significant increase in *Sb*IRP-1 level in hemocytes was also found after OsHV-1 infection, which might contribute to enhancement of the binding between *Sb*IRP-1 and the IRE of *Sb*Fn in later phases of infection. All above suggested that OsHV-1 could facilitate its own replication by interfering with the regulation of *Sb*IRP-1-*Sb*Fn pathways on iron homeostasis.

In conclusion, a full-length cDNA of *Sb*IRP-1 was identified and characterized, *Sb*IRP-1 showed highly sequential and structural conservation with other IRP1s. The interaction between *Sb*IRP-1 and the IRE of *Sb*Fn was verified by EMSA. The IRE/IRP system conservatively served on the regulation of *Sb*Fn in ark clams. When *Sb*IRP-1 was knocked down in vivo by RNAi, the accumulation level of *Sb*Fn could be detected in hemocytes by Western blot, even without significant changes to *Sb*Fn mRNA levels. OsHV-1 infection was shown to promote the protein level of *Sb*IRP-1, which might contribute to the stronger binding ability of *Sb*IRP-1 to the IRE of *Sb*Fn, blocking the translation of *Sb*Fn.

## Figures and Tables

**Table 1:** The Information of Sequences Used in this Study.

Primer Name	Primer Sequence (5′–3′)
Gene clone primer	
*Sb*IRP-F	ATGGCATGTGGATCAAACCCTTACA
*Sb*IRP-R	TCACAACATCTGCCGAATCATGTAG
Real-time quantitative PCR primers	
q*Sb*IRP-F	GGACTCGGTGTTGTCGGTTGG
q*Sb*IRP-R	GACGCAAATGCTTTGTAATGGTC
q*Sb*Fn-F	ACTCTGCCACCTCTCTTGTTCTG
q*Sb*Fn-R	TGCCAGTTATGTCTATCAGTCCA
q*Sb*RL15-F	AGACCAGACAAAGCCAGAAGAC
q*Sb*RL15-R	GCTGAAGTAAGTCCACGCATT
Vector construction primers	
*Sb*IRP-*Nco* I	CATGCCATGGGCGCATGTGGATCAAACCCTTACA
*Sb*IRP-*Xho* I	CCGCTCGAGTCAACATCTGCCGAATCATGTAG
*Sb*IRP interference	
Sense	CCAGGUCAAUCUAGAGUAUTT
Anti-sense	AUACUCUAGAUUGACCUGGTT
Negative control interference	
Sense	UUCUCCGAACGUGUCACGUTT
Anti-sense	ACGUGACACGUUCGGAGAATT
EMSA probe	
SbFn IRE	AUUUGUUUUGCUGCGUCAGUGAACGUACGGACAGCU
Mutant SbFn IRE	AUUUGUUUUGUUGCGUuuuUuuACGUACGGACAGCU
RNA-ChIP primers	
ChIP-F	AACGTACGGACAGCTTGTGA
ChIP-R	GTCTTGGTTGTGTTTGAGCCA

## Data Availability

Not applicable.
